# Spatio-Temporal Modeling of Zika and Dengue Infections within Colombia

**DOI:** 10.3390/ijerph15071376

**Published:** 2018-06-30

**Authors:** Daniel Adyro Martínez-Bello, Antonio López-Quílez, Alexander Torres Prieto

**Affiliations:** 1 Department of Statistics and Operations Research, Faculty of Mathematics, University of Valencia, 46100 Valencia, Spain; antonio.lopez@uv.es; 2 Epidemiologic Monitoring Office, Secretary of Health of the Department of Santander, Cl. 45 11-52 Bucaramanga, Colombia; atorrespri@hotmail.com

**Keywords:** disease mapping, Bayesian modeling, integrated nested Laplace approximation, relative risk

## Abstract

The aim of this study is to estimate the parallel relative risk of Zika virus disease (ZVD) and dengue using spatio-temporal interaction effects models for one department and one city of Colombia during the 2015–2016 ZVD outbreak. We apply the integrated nested Laplace approximation (INLA) for parameter estimation, using the epidemiological week (EW) as a time measure. At the departmental level, the best model showed that the dengue or ZVD risk in one municipality was highly associated with risk in the same municipality during the preceding EWs, while at the city level, the final model selected established that the high risk of dengue or ZVD in one census sector was highly associated not only with its neighboring census sectors in the same EW, but also with its neighboring sectors in the preceding EW. The spatio-temporal models provided smoothed risk estimates, credible risk intervals, and estimation of the probability of high risk of dengue and ZVD by area and time period. We explore the intricacies of the modeling process and interpretation of the results, advocating for the use of spatio-temporal models of the relative risk of dengue and ZVD in order to generate highly valuable epidemiological information for public health decision making.

## 1. Introduction

Colombia is a country located in the northwestern corner of South America. Extensive regions of the country provide good conditions for the development of vector-borne diseases such as dengue, malaria, and yellow fever, among others [1]. In 2015 and 2016, the Colombian population, as for populations of other South American countries [2,3], was exposed to the Zika virus disease (ZVD), resulting in an estimated population of 106,659 people affected up to December 2016 [4]. During the same period, the incidence of dengue infections did not vanish but rather stayed at similar levels as in previous years [5].

The ZVD epidemics in Colombia have been studied through descriptive epidemiological analyses analyse [6,7,8], ecological analyses [9,10,11,12,13], and probabilistic modeling [14,15,16].

However, epidemiological disease-mapping techniques, for example, those used for dengue disease to characterize the spatial and temporal pattern in disease risk over extended geographical regions [17,18], have not yet been applied to ZVD. Several levels of spatio-temporal modeling of dengue have been applied to continental, national, and municipal data in Brazil [19,20,21,22], Colombia [23,24,25,26,27], Ecuador [28,29], and Indonesia [30]. Until recently, most of the spatio-temporal relative risk models were developed under the Bayesian paradigm, applying the Markov chain Monte Carlo (MCMC) method. However, a new technique has emerged, providing support to modelers and epidemiologists through the use of the integrated nested Laplace approximation (INLA) [31], a fast and accurate tool for disease risk estimation.

On the basis of the modeling framework of spatio-temporal interaction effects models for relative risk developed by Knorr-Held [32], Martínez-Bello et al. [26] applied spatio-temporal risk models including covariates in Colombia at the city level using the MCMC method, while Lowe et al. [20,21,22], Wijayanti et al. [30], and Abd Naeeim & Rahman [33] applied INLA to estimate relative risk in the development of large-scale dengue warning systems in Brazil, Indonesia, and Malaysia, respectively.

The aim of this study was to model the spatio-temporal relative risk of ZVD and dengue in parallel during the period corresponding to the Zika outbreak in Colombia from October 2015 to December 2016 in one high-incidence city and one high-incidence department of Colombia, using the epidemiological week (EW) as the time measure and the census sector (city level) and municipality (departmental level) as geographic units. Our study had two specific objectives: first, to apply models describing the spatio-temporal risk of dengue and ZVD at two geographic aggregation levels, and second, to compare the risk of dengue and ZVD. For the first objective, the disease-mapping models smoothed the risk of dengue and ZVD, improving the risk visualization by area and time period and generating credible intervals of risk. For the second objective, the model detected high-risk areas of dengue and ZVD with a probability threshold defined by the data analyst. The probability estimates for relative risk permitted the formulation of approximate hypotheses for detecting areas in which the relative risk of dengue and ZVD was greater than 1 with 95% probability.

## 2. Materials and Methods

### 2.1. Zika and Dengue Data in Santander and Bucaramanga, Colombia

**Figure 1:**
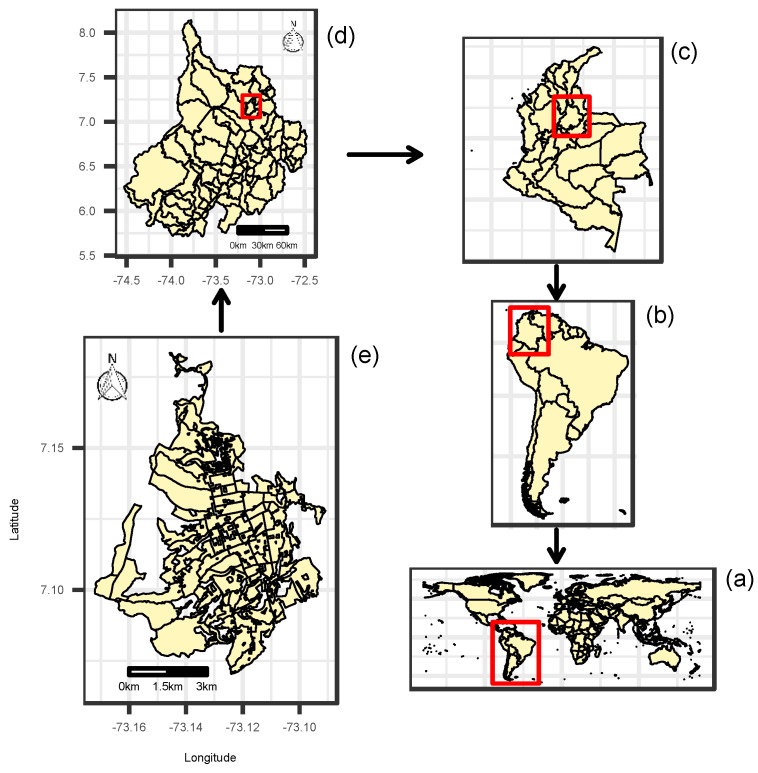
Geographical location of the study area: (**a**) world map; (**b**) South America; (**c**) Colombia; (**d**) department of Santander; (**e**) city of Bucaramanga.

Figure 1a,b shows the position of Colombia in the world and in the South American continent, respectively. Colombia (Figure 1c) is divided into administratively autonomous departments, which in turn are divided into municipalities. Figure 1d shows the department of Santander, located in northeastern Colombia, covering an area of 30,537 km${}^{2}$ and with a population of 2,071,016 people in 2016. Santander has 87 municipalities, and its administrative capital is the city of Bucaramanga (Figure 1e), which has an elevation of 959 m and which had an estimated population of 521,857 people spread over an urban area of 49 km${}^{2}$ in 2016.

Data on age, sex, and address for people with ZVD and dengue were obtained from the public health surveillance system (SIVIGILA) [34] for the period from the 42nd EW (October) of 2015 to the 52nd EW (December) of 2016. SIVIGILA is maintained by the Colombian National Institute of Health (INS) and covers nearly 11,000 health services providers, over 1117 municipalities, 32 departments, and 5 districts. The objectives of SIVIGILA are to systematically collect, analyze, interpret, update, publish, and evaluate data related with health events for the purposes of prevention and control activities in public health. SIVIGILA provides protocols for reporting each event of public health interest (EPHI) to be notified. In SIVIGILA, dengue, severe dengue, and dengue mortality are registered with the codes 210, 220, and 580, respectively, and the ZVD code is 895. We considered the case definitions obtained from SIVIGILA’s protocols for dengue and ZVD. For dengue, we considered the cases that were classified as probable, confirmed by laboratory testing and by the epidemiological nexus, as well as the dengue mortality cases, while for ZVD, we considered the cases that were classified as probable, confirmed by clinical signs and by laboratory cases, as reported to the surveillance system weekly. At Santander’s aggregation level, dengue and ZVD cases were aggregated by disease, municipality, and EW, and for the city of Bucaramanga, patients’ addresses were geocoded using the ArcGIS Online Geocoding Service and then aggregated by disease, census sector, and EW. Census sectors are geographical areas employed by the Colombian statistical office to report census data. Census sectors correspond to the aggregation of 2 to 20 census blocks [35].

### 2.2. Expected Values for ZVD and Dengue

The next step was to compute ZVD and dengue expected values, which are necessary as input for the spatio-temporal risk models. Expected values were computed using the incidence rate (disease cases per 100,000 people) by 5 year age group and sex at the departmental and city levels as follows:

$$\begin{array}{c} {\mathrm{IR}}_{kl}=\frac{{\mathrm{Cases}}_{kl}}{{\mathrm{Population}}_{kl}}\times 100,000, \end{array}$$

where IR${}_{kl}$ is the incidence rate in 5 year age groups k=1,…,K(1=[0,5),2=[5,10),…,14=[65,100)) and sex l=1,2, (1 = female, 2 = male); Cases${}_{kl}$ are the total number of ZVD or dengue cases; and Population${}_{kl}$ is the total population in 5 year age groups *k* and sex *l* for the departmental or city level over the complete study period, as obtained from the Colombian census for the 2016 population [36]. After computing IR${}_{kl}$, the expected values per small area (municipality for the departmental aggregation level or census sector for the city aggregation level) were calculated as follows:

$$\begin{array}{c} E_{i}=\sum_{k=1}^{K}\sum_{l=1}^{2}\left({\mathrm{IR}}_{kl}\times {\mathrm{Population}}_{ikl}\right), \end{array}$$

where $E_{i}$ is the expected value in small area *i* and Population${}_{ikl}$ is the census population [36] in small area *i* and 5 year age group *k* and sex *l*. Finally, the $E_{i}$ values are divided by the number of periods *t* to obtain the expected values ($E_{ij}$) by area *i* and time period *j*. At the end, four sets of expected values were obtained: Santander’s ZVD and dengue expected values and Bucaramanga’s ZVD and dengue expected values.

The ratio between the observed and the expected values of dengue and ZVD per area *i* and time period *j* is a statistic referred to as the standardized incidence ratio (SIR). The SIR is a raw estimate of the disease risk, which can be modeled by the relative risk estimation assuming a probability distribution, producing point estimates and credible intervals for the risk together with other valuable statistics, such as probability estimates of high risk per area and time period [37].

### 2.3. Spatio-Temporal Relative Risk Models

We let the observed counts of dengue or ZVD cases be $O_{ij}$, where i=1,…,n is the small area (n= 87 municipalities or n= 94 census sectors) and j=1,…,t (t= 91 EWs) denotes the temporal unit. We assume that the observed counts are Poisson distributed with mean parameter $\mu_{ij}$ as follows:

(1) $$\begin{array}{cc} O_{ij} & ∼\mathrm{Poisson}(\mu_{ij}),\\ \mu_{ij} & =E_{ij}r_{ij},\\ r_{ij} & =\exp(\eta_{ij}),\\ \eta_{ij} & =\alpha +\zeta_{i}+\gamma_{j}+\phi_{j}+\delta_{ij}, \end{array}$$

where $E_{ij}$ represents the expected values of dengue or ZVD calculated by internal or external standardization; $r_{ij}$ denotes the relative risk of dengue or ZVD by small area and EW; and $\eta_{ij}$ is a linear predictor including latent variables accounting for the spatial, temporal, and spatio-temporal dengue or ZVD risk structure. In Equation (1), α is a mean parameter, $\zeta_{i}$ accounts for the spatially structured risk pattern, $\gamma_{j}$ and $\phi_{j}$ represent the temporally unstructured and structured risk patterns, and $\delta_{ij}$ is the interaction term accounting for the spatio-temporal risk pattern. The probabilistic structure for the model parameters is

$$\begin{array}{cc} \zeta & ∼\mathrm{Normal}(0,\sigma_{\zeta }^{2}R^{-1}),\\ R & =(\rho_{\zeta }Q_{\zeta }+\left(1-\rho_{\zeta }\right)I_{\zeta }),\\ \gamma & ∼\mathrm{Normal}(0,\sigma_{\gamma }^{2}I^{-1}),\\ \phi & ∼\mathrm{Normal}(0,\sigma_{\phi }^{2}Q_{\phi }^{-1}), \end{array}$$

where the ζ vector represents conditional autorregressive (CAR) Leroux spatially structured effects, representing the joint distribution of the structured spatial pattern, normally distributed with zero mean vector and variance–covariance matrix $\sigma_{\zeta }^{2}R^{-1}$. The R matrix follows Leroux et al. [38], formulated by Ugarte et al. [39,40], where $\rho_{\zeta }$ is a smoothing parameter estimated from the data, $Q_{\zeta }$ is an n×n proximity matrix, and $I_{\zeta }$ is an n×n identity matrix. The $Q_{\zeta }$ matrix contains values of 1 where the area *i* is neighbor of area $i^{\prime }$ and 0 otherwise. The γ vector represents the joint distribution of the unstructured temporal effects, normally distributed with zero mean vector and variance–covariance matrix $\sigma_{\gamma }^{2}I_{\gamma }^{-1}$, where $\sigma_{\gamma }^{2}$ is a variance parameter and $I_{\gamma }$ is a t×t identity matrix. The ϕ vector is the joint distribution of the structured temporal effects, normally distributed with zero mean vector and variance–covariance matrix $\sigma_{\phi }^{2}Q_{\phi }^{-1}$, where $\sigma_{\phi }^{2}$ is a variance parameter and $Q_{\phi }$ is a t×t random walk 1 or 2 (RW1 or RW2) matrix. The joint distribution of the spatio-temporal interaction-effects vector $\delta^{\prime }=\left[\delta_{11},\delta_{12}\ldots ,\delta_{nt}\right]$ is normally distributed with mean zero vector and variance–covariance matrix defined by one of four interaction types as follows:

(2) $$\begin{array}{cc} \delta & ∼\mathrm{Normal}(0,\sigma_{\delta }^{2}{\left(I_{\gamma }⊗I_{\zeta }\right)}^{-1}), \end{array}$$

(3) $$\begin{array}{cc} \delta & ∼\mathrm{Normal}(0,\sigma_{\delta }^{2}{\left(Q_{\phi }⊗I_{\zeta }\right)}^{-1}), \end{array}$$

(4) $$\begin{array}{cc} \delta & ∼\mathrm{Normal}(0,\sigma_{\delta }^{2}{\left(Q_{\zeta }⊗I_{\phi }\right)}^{-1}), \end{array}$$

(5) $$\begin{array}{cc} \delta & ∼\mathrm{Normal}(0,\sigma_{\delta }^{2}{\left(Q_{\phi }⊗Q_{\zeta }\right)}^{-1}), \end{array}$$

where for all types of interaction effects, $\sigma_{\delta }^{2}$ is a variance parameter and ⊗ is the Kronecker product of two matrices. We follow the Knorr-Held [32] interaction-effects taxonomy. Equation (2) shows the type I interaction effects δ with structure matrix $\left(I_{\gamma }⊗I_{\zeta }\right)$, representing an unstructured interaction effect, where $I_{\gamma }$ is a t×t identity matrix and $I_{\zeta }$ is an n×n identity matrix. Equation (3) displays the type II interaction effects δ with structure matrix $\left(Q_{\phi }⊗I_{\zeta }\right)$ modeling a temporal interaction effect, where $Q_{\phi }$ is t×t RW1 or RW2 matrix and $I_{\zeta }$ is as defined above. Equation (4) shows the type III interaction effects δ with structure matrix $\left(Q_{\zeta }⊗I_{\gamma }\right)$ representing spatial interaction effects, where $Q_{\zeta }$ is the n×n proximity matrix defined above and $I_{\gamma }$ is a t×t matrix previously defined. Finally, Equation (5) shows the type IV interaction effects δ with structure matrix $\left(Q_{\phi }⊗Q_{\zeta }\right)$, defining inseparable spatio-temporal interaction effects, where $Q_{\phi }$ is a t×t RW1 or RW2 matrix and $Q_{\zeta }$ is an n×n proximity matrix.

### 2.4. Inference

The Bayesian inference for the spatio-temporal models is currently developed by applying MCMC methods through conditional probability distributions of the model parameters; however, we followed the INLA [31] technique to fit the spatio-temporal interaction effects models to the data, using the INLA package downloaded from www.r-inla.org and R software, version 3.3 [41]. We followed the constraints specification of Goicoa et al. [42] to address the parameter identifiability issues in the interaction terms. The hyperprior specifications for the mean parameter (α), the variance hyperparameters ($\sigma_{\zeta }^{2}$, $\sigma_{\gamma }^{2}$, $\sigma_{\phi }^{2}$, and $\sigma_{\delta }^{2}$), and the smoothing parameter ($\rho_{\zeta }$) are as follows:

$$\begin{array}{cc} \alpha & ∼\mathrm{Normal}(0,1000),\\ \rho_{\zeta } & ∼\mathrm{Uniform}(0,1),\\ \sigma_{\zeta },\sigma_{\gamma },\sigma_{\phi },\sigma_{\delta } & ∼\mathrm{Uniform}(0,\infty ). \end{array}$$

Model selection was on the basis of the analysis of the deviance, the effective number of parameters, the Watanabe–Akaike information criterion (WAIC) by Watanabe [43], and the logarithmic score (LS) by Gneiting and Raftery [44] and implemented by Ugarte et al. [40]. The selected final model corresponded to the model displaying the lowest WAIC and LS.

## 3. Results

### 3.1. Exploratory Data Analysis

First, we obtained from the Santander’s SIVIGILA database a total of 10,051 ZVD cases (63.1% females and 36.9% males) and 7891 dengue cases (48.6% females and 51.4% males), while Bucaramanga’s database included 3662 ZVD cases (61.2% females and 38.8% males) and 2470 dengue cases (49.3% females and 50.7% males).

**Figure 2:**
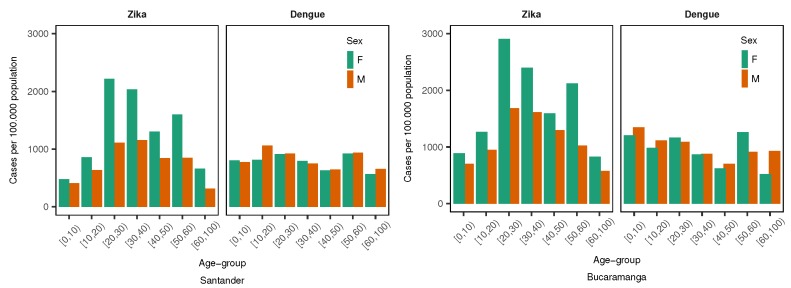
Incidence rate of Zika virus disease (ZVD) and dengue (cases per 100,000 people) by 10 year age group and sex in the department of Santander and the city of Bucaramanga from October 2015 to December 2016.

Then, using the number of dengue and ZVD cases by age group and sex, along with the populations of the department of Santander and the city of Bucaramanga, we calculated the incidence rate (cases by 100,000 people) by age and sex. Figure 2 displays the incidence rate by 10 year age group and sex for dengue and ZVD at the departmental (Santander) and city (Bucaramanga) levels. In general, incidence rates were higher at the city level than in the department as a whole. Both geographic levels presented a similar incidence pattern for dengue and ZVD. Dengue disease incidence was slightly higher in younger than older people but was very similar for women and men, while ZVD incidence was higher in the age range of 20 to 65 years for women than for men. The incidence rates were used to generate the expected values per area and time period; therefore, the study used internal standardization. Thereafter, the expected values for dengue and ZVD at the departmental and city levels per area and EW were combined with the observed values to produce the SIR values per area and time period.

**Figure 3:**
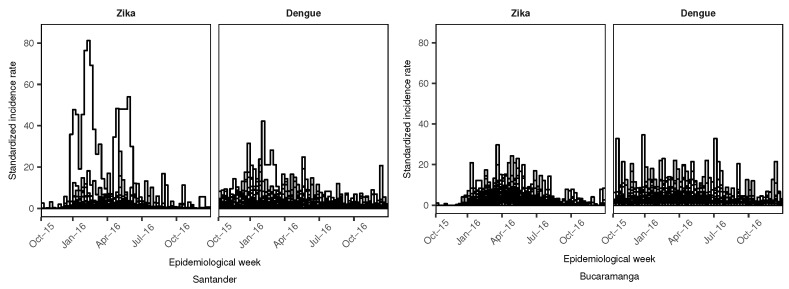
Longitudinal profiles of the standardized incidence ratio (SIR) of dengue and Zika virus disease (ZVD) by municipality (department of Santander) and census sector (city of Bucaramanga) from October 2015 to December 2016.

Figure 3 presents the longitudinal profiles of the SIR by aggregation area for dengue and ZVD during the study period. For the department of Santander, the ZVD SIR profiles showed values of more than 40 units in two municipalities, but in general, most of the municipalities did not surpass SIR values of 20 units. For dengue, the SIR was consistently under 20 units throughout the study period in most municipalities, with two spikes in incidence in December 2015 and May 2016. For the city of Bucaramanga, the SIR for ZVD was under 20 units during the outbreak between January 2016 and July 2016, and dengue had a constant SIR of less than 20 units during the study period.

**Figure 4:**
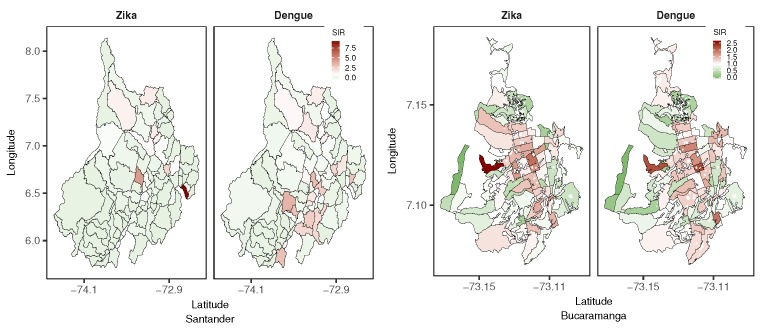
Accumulated standardized incidence ratio (SIR) maps of dengue and Zika virus disease (ZVD) in Santander and Bucaramanga from October 2015 to December 2016.

The SIR could also be accumulated in the study period and mapped by area by employing a choropleth map to visualize SIR patterns. Figure 4 shows maps of the accumulated SIR for dengue and ZVD by aggregation area in Santander and Bucaramanga for the study period. The accumulated ZVD SIR in Santander showed that few municipalities had high SIR levels in the northern, central, and eastern areas of the department, while high values for the accumulated dengue SIR were presented in the southern municipalities of the department. In contrast, at the city aggregation level, high values for the accumulated ZVD and dengue SIRs were apparent in the central census sectors of the city, displaying similar distribution patterns for both diseases.

### 3.2. Model Findings

Table 1 displays the selection criteria statistics for the spatio-temporal relative risk models of dengue and ZVD for Santander and Bucaramanga. At the departmental aggregation level, and using the WAIC and the LS, the selected model contained type II interaction effects (temporal effects) for both dengue (WAIC of 7413.6 and LS of 3738.7) and ZVD (WAIC of 4562.9 and LS of 2362.7). At the city aggregation level, the best model corresponded to that with type IV interaction effects (spatio-temporal inseparable effects) for dengue (WAIC of 8010.4 and LS of 4005.7) and ZVD (WAIC of 7804.1 and LS of 3904.1).

Table 2 presents the smoothing parameters ($\rho_{\zeta }$) of the spatially structured random effects and the standard deviation hyperparameters corresponding to the final selected models for Santander and Bucaramanga. At the departmental level, the smoothing parameters displayed posterior mean values of 0.61 and 0.50 for ZVD and dengue, respectively, which denoted moderate spatially structured effects. We observed similar results at the city level, where the posterior means of the smoothing parameters were 0.55 and 0.49 for ZVD and dengue, respectively. The standard deviation hyperparameters at the departmental level were higher for ZVD than for dengue, and the small standard deviation of the temporally structured effects ($\sigma_{\phi }$) denoted the small variability of ϕ. At the city level, $\sigma_{\phi }$ also showed small variability for all the hyperparameters.

**Figure 5:**
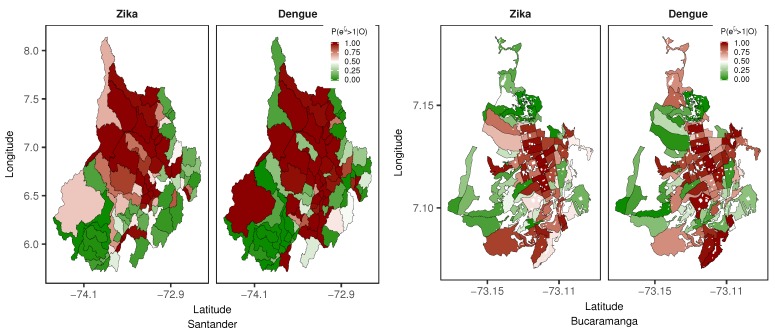
Probability of spatial structured effects greater than 1 $\left(P(\exp\left(\zeta_{i}\right)>1|O)\right)$ for department of Santander and city of Bucaramanga from October 2015 to December 2016.

Using the selected models for dengue and ZVD in Santander (model with type II interaction effects) and Bucaramanga (model with type IV interaction effects), the probability of the structured spatial risk pattern being greater than 1 given the observed disease counts (P(exp($\zeta_{i}>1|O$)) of the entire study period was mapped and is shown in Figure 5. What can this probability indicate to epidemiologists, biostatisticians, and public health officials? The mapped probability provides estimates of the areas at the departmental and city levels for which a high risk of dengue or ZVD was shared. From Figure 5, in the department of Santander, the red areas reveal the municipalities with probabilities close to 1, displaying a high risk with a clustered pattern; thus, for ZVD, the northern and western municipalities presented the spatially clustered high-risk pattern, while for dengue, the north and south municipalities showed the spatially clustered high-risk pattern. At the city level, the distributions of high-risk spatial clusters (probability close to 1) of census sectors were similar for both dengue and ZVD and were concentrated in the central parts of the city, following the pattern displayed by the SIR maps in Figure 4.

**Figure 6:**
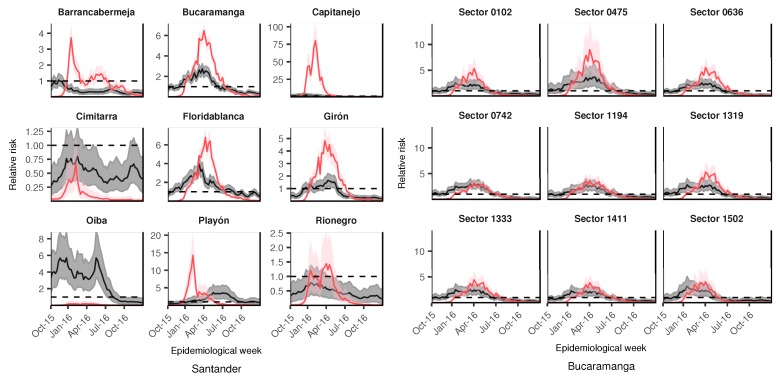
Selected longitudinal profiles of the posterior means and 95% credible intervals for the relative risk of dengue (gray shadow) and Zika virus disease (ZVD) (pink shadow) for department of Santander and city of Bucaramanga from October 2015 to December 2016. Dashed lines correspond to relative risk equal to 1.

In addition to the spatial structured effects, the relative risk per area and time period could be obtained from the final selected spatio-temporal models. For the department of Santander, Figure 6 shows the longitudinal profiles of relative risk for dengue (gray) and ZVD (pink), displaying the posterior mean and 95% credible intervals for the selected municipalities. Before examining the plot, we warn the reader that different scales for the relative risk are displayed for the selected municipalities, because the high variability of the risk profiles from municipality to municipality would make it difficult to visualize the trend in several areas. The municipalities were selected on the basis of the high probability of the spatial structured pattern from Figure 5. Three nearby municipalities (Bucaramanga, Girón, and Florida) revealed similar risk patterns for both diseases, showing that the ZVD outbreak closely followed the dengue outbreak in the first semester of 2016, while municipalities such as Cimitarra and Rionegro showed a low risk for dengue and ZVD, and Capitanejo displayed the highest relative risk of all the municipalities in the department. At the city level (Bucaramanga), some selected census sectors with probabilities close to 1 for the spatial structured effects did not reveal the high variability presented at the departmental level, showing the high risk of ZVD in close connection with that of dengue, and with the ZVD risk being higher than the dengue risk for the selected census sectors.

**Figure 7:**
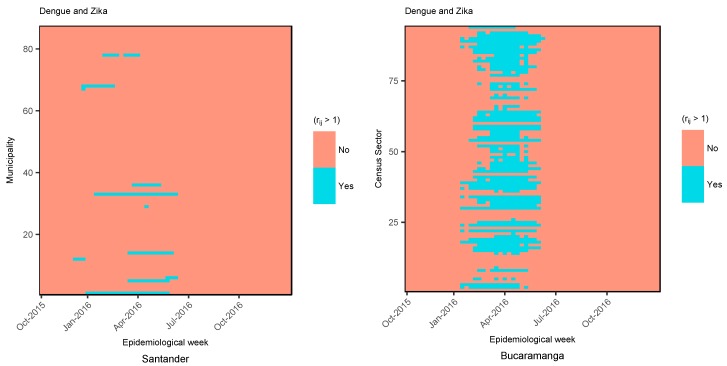
Heatmap of the relative risk greater than 1 ($r_{ij}$ > 1) with 95% probability for dengue and Zika virus disease (ZVD) in the department of Santander and the city of Bucaramanga from October 2015 to December 2016.

In the search for a combined risk representation of dengue and ZVD, Figure 7 explores the spatio-temporal representation of relative risk being greater of 1 with 95% probability for both diseases, that is, the areas in which the 95% credible intervals of the risk for dengue and ZVD were higher than 1. For this purpose, the heatmap for Santander represents the municipalities with risk greater than 1 with 95% probability, following the selected models for dengue and ZVD in Santander, and exposes the strictly temporal risk pattern by municipality, with few municipalities sharing a relative risk of greater than 1 with 95% probability. The shared relative risk greater than 1 with 95% probability displayed a somewhat different pattern at the municipality level. For the ZVD outbreak period (January to July 2016), almost all the census sectors demonstrated a relative risk greater than 1 with 95% probability for both diseases, taking into account that the risk representation followed the selected final models for dengue and ZVD corresponding to the type IV interaction-effects model (spatio-temporal inseparable interaction).

**Figure 8:**
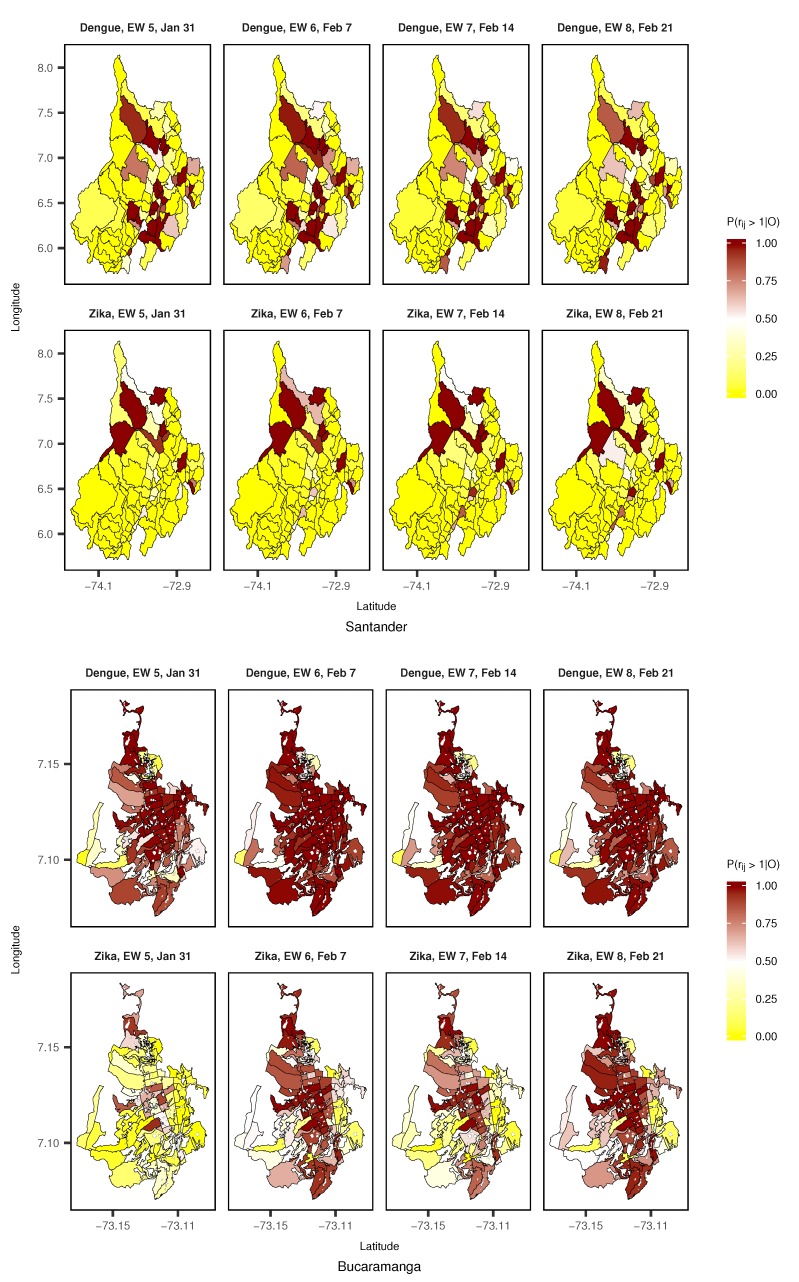
Evolution of the probability of dengue and Zika virus disease (ZVD) relative risk greater than 1 given the observed cases (P($r_{ij}>1|O$)) for selected epidemiological weeks (EWs) in January and February 2016 in the department of Santander and the city of Bucaramanga.

Not only could the relative risk be presented as shown in Figure 6 using longitudinal posterior mean profiles and credible intervals, but it could also be mapped directly (posterior mean or selected percentiles) or be represented by the probability of a relative risk greater than 1 given the observed cases counts (P($r_{ij}>1|O$)). Figure 8 exhibits maps of the probability of relative risk greater than 1 for dengue and ZVD in Santander and Bucaramanga from the fifth to eighth EWs of 2016 (the period associated with the rapid dissemination of ZVD at the departmental and city levels). For those EWs, the municipalities with a high probability of relative risk greater than 1 for dengue in Santander were revealed to be the central northern and southern municipalities, while for ZVD, the high-probability municipalities were in the northwestern region of the department. In Bucaramanga, almost all census sectors displayed a high probability of relative risk greater than 1 for dengue, and many census sectors also revealed a high probability for ZVD during the same time period across the city.

## 4. Discussion

The present study applied parallel spatio-temporal interaction-effects models of relative risk to ZVD and dengue data at two geographic levels of aggregation, first at the departmental level (municipality aggregation), and second at the city level (census sector aggregation). The aim was to provide risk estimates for arboviral diseases, elucidating the risk transmission dynamics of dengue and ZVD.

We fitted the spatio-temporal models using INLA, which is a fast and accurate numerical method to develop Bayesian analysis.

The within-sample predictive measures WAIC and LS were the information criteria applied to select the final models at the departmental and city levels. The model selection was based on the models showing the lowest values for the information criteria. At the departmental level, the final selected model included type II (temporal interaction) spatio-temporal interaction effects, CAR Leroux structured spatial effects, and RW1 structured temporal effects. Selecting the type II model for inference implied that the dengue or ZVD risk in one municipality was highly associated with risk in the same municipality during the preceding EW. At the city level, the final selected model included type IV (inseparable) spatio-temporal interaction effects, CAR Leroux structured spatial effects, and RW1 structured temporal effects. The selection of the type IV model implied that the risk of dengue or ZVD in one census sector was highly associated not only with its neighboring census sectors in the same EW, but also with its neighboring sectors in the preceding EW.

The information concerning the relative risk obtained from the models could be presented in many ways. We provide longitudinal profiles of relative risk (posterior mean and 95% credible intervals) and maps of the high-risk probability by municipality or census sector. In addition, we represent the joint risk of dengue and ZVD using 95% credible intervals by means of creating a categorical variable representing high risk when the lower limit of the 95% credible interval was greater than 1, identifying municipalities or census sectors at a high risk with 95% probability for dengue and ZVD. At the departmental level, we distinguished 11 municipalities displaying a high risk for dengue and ZVD, while at the city level, almost all the census sectors showed joint high risk for both diseases.

It is difficult to compare the findings in our study with other results in the dengue and ZVD literature, because a parallel risk assessment of ZVD and dengue under a spatio-temporal setting is not current research practice, perhaps because of the recent emergence of ZVD in the affected countries. For the Colombian case, Krystosik et al. [45] associate perceived risk factors such as proximity to standing water, canals, poverty, localized violence, and military migration, with density maps of dengue, ZVD, and Chikungunya virus disease (CVD) cases at the city level, using spatial point pattern methods. Krystosik et al. identified suitable areas for disease transmission, ignoring the temporal nature of the data, which our study considers, although we did not consider an association between covariates for environmental or socioeconomic factors and dengue or ZVD.

Chien et al. [46] published another case study using Colombian data, exploring a quasi-Poisson spatial nonlinear model to investigate the association among weekly ZVD infection and lagged effects of weather variables, using the department as the spatial unit, from January 2015 to December 2016. The modeling results suggest that mean humidity, total rainfall, and the maximum temperature can help to predict a ZVD infection outbreak at least 3 months in advance, suggesting the model could be applicable in the development of a ZVD surveillance system by public health authorities. The study of Chien et al. also modeled the relative risk of ZVD; however, the relative risk was based on non-smoothed estimates. In addition, they ignored the spatio-temporal nature of the outcome variable while investigating the lagged effects of the covariates, and thus their results were based on a fixed-in-time outcome variable, and the risk association between areas mediated by the spatial nature of the data was ignored. They also used a spatial aggregation unit much larger than that presented in our study, and finally, they analyzed only ZVD, not dengue or CVD.

For the city of Bahía de Caráquez in Ecuador (a country neighboring Colombia), Stewart-Ibarra et al. [47] associated the degree of psychological distress with the presence of dengue, CVD, and Zika infections in a suburban community after the 2016 earthquake. The study followed a survey methodology, without considering the temporal or spatial nature of the data and collapsing all three arboviral diseases studied into one composite variable. Thus, this paper did not investigate the possible differences in transmission for the diseases.

In the course of the concurrent disease outbreaks of CVD and ZVD in French Polynesia and the French West Indies between 2013 and 2016, Riou et al. [48] modeled the joint transmission of both diseases using a Bayesian time-dependent susceptible–infectious–recovered (TSIR) framework [49] including information on weather conditions (temperature and precipitation). This study is interesting because the findings can be related to our results. The authors found that after controlling for locality and weather conditions, the difference in transmission between the two viruses was minimal, suggesting that the epidemic dynamics were more dependent on factors related to the mosquito abundance and local environmental and socioeconomic conditions than a result of the intrinsic characteristics of the viruses. In addition, the authors modeled the risk between ZVD and CVD, considering the between-island association, which we also accounted for in our modeling framework.

In Brazil, one of the American countries most affected by the 2015–2016 ZVD epidemic, Aguiar et al. [50] assessed the potential spatial risk of ZVD and CVD using a maximum entropy (MaxEnt) approach, estimating the optimal conditions for infection on the basis of the disease occurrence and environmental and social factors for 2015–2016 at the municipality level. A local empirical Bayesian (LEB) analysis was performed using the cases of ZVD and CVD reported by municipalities, and then, the municipalities with LEB rates greater than zero were included in the MaxEnt analysis. The analyses provided joint risk maps of ZVD and CVD. Although the study by Aguiar et al. included covariates for the analysis, the authors also ignored the spatio-temporal nature of the data, such as those described in the previous studies shown in the current section.

The spatio-temporal models of relative risk shown here are available to study dengue and ZVD transmission, acting as predictive models to help public health officers anticipate the time and place the next outbreak of ZVD or other emerging arboviral diseases will occur [51]. Identifying high-risk areas of vector-borne diseases at a small geographic resolution using within-sample predictive models or short-time-ahead, out-of-sample predictive models supports the control and prevention of vector-transmitted diseases, which is one the advantages of the spatio-temporal methods applied in the present study [52]. The modeling approach in our study could easily be combined with covariates originating from vectorial ecology, socioeconomic, and infrastructural data, improving the information generated from the modeling exercise, as demonstrated for dengue by Wijayanti et al. [30]. Such studies could clarify the role of factors such as land cover, meteorological, and socioeconomic determinants of arboviral diseases [53].

This study presents some limitations. For dengue data, it is well known that underreporting could reach almost 70%, because many affected people do not approach health services and some of the patients attending the health services are not correctly diagnosed [54]. There are few ZVD studies addressing the quality of the reporting and diagnosis; however, one study from the National Institute of Health in Colombia, carried out in a hyperendemic ZVD town in Colombia, described around 49% of ZVD underreporting for people detected in a community active search of ZVD cases and 81% of ZVD underreporting for people detected in a health service active search [55]. The intrinsic characteristics of the data make the results of the study prone to the same inaccuracies as the event reports produced by the public health surveillance system, because we used exactly the same data registered and analyzed by the system. For instance, the system’s event notification depends on the medical doctor’s ability to recognize and register the notifiable event, only using laboratory testing for severe dengue cases or for pregnant women and children with ZVD. Even with the surveillance system underreporting ZVD and dengue, the spatio-temporal risk estimates would complement the current information generated by the system.

To end our discussion, we need to mention that we modeled ZVD and dengue independently before combining the risk to obtain a joint risk estimation. However, a framework of spatial joint models of relative risk exist [37] and could be explored to include several diseases at the same time. In addition, it would be possible to fit longitudinal spatio-temporal joint models, providing a full structure for estimation of relative risk, although the implementation is not straightforward. A following step of our research is to test joint spatial and spatio-temporal models of dengue, ZVD, CVD, and other emerging vector-borne infectious diseases.

## 5. Conclusions

We explored a statistical method to express the risk of ZVD and dengue using spatio-temporal models of relative risk, providing a coherent, systematic, and structured approach to model implementation, applying the modern INLA technique for Bayesian parameter estimation. We argue that the spatio-temporal risk framework has advantages for public health officers, epidemiologists, biostatisticians, and data analysts involved in controlling vector-borne infectious diseases, corresponding with fast and accurate risk estimation (provided that good quality data are available), risk smoothing for trend visualization, and the generation of credible intervals expressing the risk probability, which could be portrayed in spatial or temporal features. We also observed shared areas of ZVD and dengue high risk with high probability, given a probability threshold imposed by the data analyst. We searched the literature for parallel modeling of dengue and ZVD risk, detecting a lack of spatio-temporal risk models for arboviral diseases. This study thus presents a helpful statistical method as a tool for decision making in preventing and controlling vector-borne emergent diseases.

## Figures and Tables

**Table 1:** Selection statistics (deviance, effective number of parameters (p${}_{\mathrm{eff}}$), Watanabe Akaike information criterion (WAIC), and logarithmic score (LS)) from the spatio-temporal interaction-effects models of Zika virus disease (ZVD) and dengue ($\eta_{ij}=\alpha +\zeta_{i}+\gamma_{j}+\phi_{j}+\delta_{ij}$) and random walk 1 (RW1) temporally structured effects for the department of Santander and the city of Bucaramanga from October 2015 to December 2016.

	Zika				Dengue			
	Deviance	p${}_{\mathrm{eff}}$	WAIC	LS	Deviance	p${}_{\mathrm{eff}}$	WAIC	LS
	**Department of Santander**							
No interaction	6290.6	115.7	6592.6	3307.4	8308.4	112.7	8490.5	4236.5
Type I	4222.8	630.5	4855.0	4570.9	7278.4	623.9	7934.2	4156.7
Type II	4166.2	410.4	**4562.9**	**2362.7**	6979.7	435.3	**7413.6**	**3738.7**
Type III	4247.6	589.1	4856.3	4147.4	7314.4	589.0	7968.7	4143.6
Type IV	4206.4	382.1	4593.7	2437.6	7044.8	408.1	7470.9	3764.0
	**City of Bucaramanga**							
No interaction	7852.1	105.0	7970.1	3985.3	7938.5	93.9	8042.5	4021.4
Type I	7659.3	275.0	7949.3	3980.8	7680.3	318.2	8018.5	4014.6
Type II	7537.5	263.7	7808.2	3907.3	7841.2	165.6	8024.2	4012.8
Type III	7653.2	247.7	7913.6	3961.5	7865.6	159.9	8040.7	4021.2
Type IV	7576.6	218.7	**7804.1**	**3904.1**	7839.7	155.8	**8010.4**	**4005.7**

**Table 2:** Standard deviation hyperparameters of the interaction-effects models for the relative risk of Zika virus disease (ZVD) and dengue from October 2015 to December 2016. Posterior mean, standard deviation (SD), and 2.5%, 50%, and 97.5% percentiles.

	Zika					Dengue				
	Mean	SD	2.5%	50%	97.5%	Mean	SD	2.5%	50%	97.5%
	**Department of Santander**									
$\rho_{\zeta }$	0.61	0.17	0.26	0.63	0.90	0.50	0.18	0.17	0.50	0.84
$\sigma_{\zeta }$	2.36	0.33	1.80	2.34	3.05	2.04	0.32	1.49	2.02	2.73
$\sigma_{\gamma }$	0.32	0.04	0.24	0.31	0.41	0.11	0.02	0.08	0.11	0.16
$\sigma_{\phi }$	0.06	0.04	0.01	0.05	0.16	0.04	0.02	0.01	0.04	0.09
$\sigma_{\delta }$	0.36	0.02	0.31	0.36	0.40	0.23	0.01	0.20	0.23	0.25
	**City of Bucaramanga**									
$\rho_{\zeta }$	0.55	0.20	0.16	0.55	0.89	0.49	0.19	0.15	0.49	0.84
$\sigma_{\zeta }$	0.48	0.07	0.36	0.48	0.64	0.52	0.08	0.39	0.51	0.68
$\sigma_{\gamma }$	0.42	0.06	0.33	0.42	0.54	0.19	0.04	0.13	0.18	0.27
$\sigma_{\phi }$	0.07	0.06	0.01	0.06	0.25	0.07	0.04	0.02	0.07	0.16
$\sigma_{\delta }$	0.17	0.02	0.14	0.17	0.21	0.10	0.02	0.07	0.10	0.14

## Abbreviations

The following abbreviations are used in this manuscript:

CI	Credible Intervals
CAR	Conditional Autorregressive
DANE	Departamento Nacional de Estadística
EW	Epidemiological Week
INLA	Integrated Nested Laplace Approximation
IR	Incidence Rate
LEB	Local Empirical Bayes
LS	Logarithmic Score
RW1	Random Walk 1
RW2	Random Walk 2
SD	Standard Deviation
SIR	Standardized Incidence Ratio
SIVIGILA	Sistema de Vigilancia en Salud Pública (Public Health Surveillance System)
TSIR	Time-dependent Susceptible-Infectious-Recovered
WAIC	Watanabe-Akaike Information Criterion
ZVD	Zika Virus Disease
